# Mitochondrial Transcription Factor A added to Osteocytes in a Stressed Environment has a Cytoprotective Effect

**DOI:** 10.7150/ijms.45335

**Published:** 2020-05-23

**Authors:** Shusuke Ueda, Miyako Shimasaki, Toru Ichiseki, Hiroaki Hirata, Norio kawahara, Yoshimichi Ueda

**Affiliations:** 1 Department of Orthopaedic Surgery, Kanazawa Medical University, Daigaku 1-1, Uchinada-machi, Kahoku-gun, Ishikawa 920-0293, Japan.; 2 Department of Pathology 2, Kanazawa Medical University, Daigaku 1-1, Uchinada, Kahoku-gun, Ishikawa, 920-0293, Japan.

**Keywords:** mitochondrial function, mitochondrial transcription factor A (TFAM), oxidative injury, osteocytic cell necrosis

## Abstract

The main precipitant of glucocorticoid-associated femoral head osteonecrosis is widely accepted to be an ischemic-hypoxic event, with oxidative stress also as an underlying factor. Mitochondrial DNA is more vulnerable to oxidative injury than the nucleus, and mitochondrial transcription factor A (TFAM), which plays roles in its function, preservation, and regulation is being increasingly investigated. In the present study we focused on the impact of TFAM on the relation between the oxidative injury induced by the addition of glucocorticoid to a hypoxic environment and osteocytic cell necrosis. Using cultured osteocytes MLO-Y4 in a 1% hypoxic environment (hypoxia) to which 1µM dexamethasone (Dex) was added (Dex(+)/hypoxia(+)), an immunocytochemical study was conducted using 8-hydroxy-2'-deoxyguanosine (8-OHdG), an index of oxidative stress, and hypoxia inducible factor-1α (HIF-1α), a marker of hypoxia. Next, after adding TFAM siRNA, TFAM knockdown, cultured for 24h, and mitochondrial membrane potential were measured, they were stained with ATP5A which labels adenosine triphosphate (ATP) production. Dex was added to MLO-Y4 to which TFAM had been added, and cultured for 24h in hypoxia. The ratio of dead cells to viable cells was determined and compared. Enhanced expression of 8-OHdG, HIF-1α was found in osteocytes following the addition of glucocorticoid in a hypoxic environment. With TFAM knockdown, as compared to normoxia, mitochondrial function significantly decreased. On the other hand, by adding TFAM, the incidence of osteocytic cell necrosis was significantly decreased as compared with Dex(+)/hypoxia(+). TFAM was confirmed to be important in mitochondrial function and preservation, inhibition of oxidative injury and maintenance of ATP production. Moreover, prevention of mitochondrial injury can best be achieved by decreasing the development of osteocytic cell necrosis.

## Introduction

Although glucocorticoids can be extremely effective therapeutic agents, their diverse side effects are widely known and feared. Glucocorticoid-induced osteonecrosis is one such condition that assumes an intractable course once it occurs. It is widely known that the cause of glucocorticoid-associated femoral head osteonecrosis is an ischemic-hypoxic event. Since the establishment of prophylactic measures and elucidation of the underlying pathophysiology are important issues, various investigations relying on animal experiments have been conducted 1,2, despite which the details of the underlying pathogenetic mechanisms remain elusive. One limitation of research using animal models is that diverse environmental factors may become intricately intertwined with each other in a complex fashion. This may influence and even change the nature of the condition being studied, thereby further interfering with elucidation of the pathophysiological mechanisms. In other words, grasping the underlying pathophysiology in only a single dimension is insufficient. Meanwhile in vitro it is possible to verify and examine the occurrence of injury at the cellular level. Moreover, by using cultured cells in a hypoxic environment to which glucocorticoid has been added, it is possible to create a state similar to that seen in an osteonecrosis animal model, with recently such in vitro studies being increasingly reported 3-5. Oxidative stress has been implicated in the pathogenesis of this condition, which has prompted the prophylactic use of antioxidants in a number of studies 6,7. Peroxidation has also been demonstrated to exert an adverse effect on mitochondrial DNA *in vivo* 8.

Mitochondrial DNA is more susceptible to oxidative injury than the nucleus, and when mitochondrial DNA sustains oxidative injury mitochondrial genome abnormalities are induced. In this way cellular survival and function become severely threatened. In contrast, mitochondrial transcription factor A (TFAM) is known to promote the stability and preservation of mitochondrial DNA, and is itself said to be applicable to the determination of the number of mitochondria and their function, mitochondrial DNA repair, and moreover to influence cell death due to mitochondrial DNA injury 9, 10. Various studies have been conducted on mitochondria that have sustained major oxidative injury. But similar such studies focusing on mitochondria and TFAM in bone-related conditions, in particular, osteonecrosis, are limited to only one in which a decrease in intraosseous TFAM was documented in domestic rabbits administered glucocorticoids 11. TFAM has been investigated more for the therapy and prevention of cardiovascular diseases 12-15. And since its efficacy in this context has shown promise, it is possible that it might exert a similar beneficial effect on osteocytes as well. This hope prompted us to investigate the role of TFAM in osteocytes using cultured osteocytes and TFAM knockdown, as well as determining the incidence of oxidative injury and osteocytic cell necrosis due to forced expression of TFAM in osteocytes administered glucocorticoids in a hypoxic environment.

## Materials and Methods

### Cell culture

MLO-Y4 (kerafast, Boston, USA) murine cultured osteocytic cells, which have been used previously, were cultured 16. The cells were plated on type I collagen-coated dishes (BD Biosciences, Bedford, USA) and cultured in α-minimal essential medium (α-MEM) supplemented with 2.5% (v/v) FBS, 2.5% (v/v) FCS, streptomycin (100 μg/ml) and penicillin(100units/ml) 17. Then for the hypoxia experiments, the cells were incubated for 24h in a CO_2_/tri-gas incubator (Astec, Fukuoka, Japan) set at a mixture of 5% (v/v) CO_2_ and 1% (v/v) O_2_ balanced with N_2_ 5.

### Cell viability assay

MLO-Y4 cells seeded in a type I collagen-coated 4-chamber culture slide (BD Biosciences, Bedford, USA) were cultured overnight. It has been reported that osteocytic cells in the osteonecrosis animal model in which they are cultured in a hypoxic environment to which glucocorticoid is added share the same environment as their natural *in vivo* one 5. To conduct a study under the same conditions, cells were exposed under normoxia (20% O_2_) or hypoxia (1% O_2_) in the presence or absence of 1μM dexamethasone (Dex) (MSD, Tokyo, Japan) for 24 hours (Dex(-)/normoxia, Dex(-)/hypoxia(+), Dex(+)/normoxia, Dex(+)/hypoxia(+)) 3,5. In addition, 100nM TFAM (LifeSpan BioSciences, Seattle, USA) was added to Dex(+)/hypoxia(+) and cultured for 24h (TFAM(+)) 13,14. Viability assays were then performed using an Apoptotic/Necrotic Cells Detection Kit (PromoKine, Heidelberg, Germany) according to the manufacturer's instructions, and the percentages of apoptotic/necrotic cells relative to the total cell number were determined. In the viability assays, apoptotic cells can be detected by staining with fluorescein-labeled annexin V (green fluorescence) and necrotic cells by that with Ethidium homodimer III, a highly positively charged nucleic acid probe, which is impermeant to live cells and early apoptotic cells, but stains necrotic cells and late apoptotic cells (entering into secondary necrosis) with red fluorescence. Fluorescence-positive cells were evaluated by phase contrast and fluorescence (470 nm and 530nm LED modules) microscopy using Axiovert.A1 FL-LED (Carl Zeiss, Jena, Germany).

### Knockdown analysis using siRNAs

Since TFAM is also present intracellularly in the usual state 18, siRNA was prepared and used to confirm the performance of osteocytic cells after TFAM knockdown. MLO-Y4 cells were grown in MEM Alpha Minimum essential Medium (Thermo Fisher Scientific, Waltham, Massachusetts, U.S.A.) at 37 °C under 5% CO_2_/ 95% air. RNA Interference-siRNA targeting TFAM as well as non-targeting controls were purchased from InvitrogenTM (Stealth siRNA technology; for siRNA sequences, see **Table 1**). SiRNA was transfected into MLO-Y4 using Lipofectamine RNA iMAX (InvitrogenTM) at a final concentration of 100nM. 72h post transfection, MLO-Y4 were growth-arrested by replacing the transfection medium with serum-free DMEM supplemented with 2mM L-glutamine.

### Mitochondrial membrane potential

To determine whether mitochondria were preserved, mitochondrial membrane potentials were measured. Cultured cells were stained with Mito Tracker Red CMXRos (Therno Fisher Scientific, MA, U.S.A) at a final concentration of 200nM in culture medium at 37°C under 20% O_2_ and 5% CO_2_ for 30 minutes. Cells were then fixed with 4% paraformaldehyde; nuclei were stained with 4', 6-diamidino-2-phenylindole (DAPI).

Mitochondrial DNA sustained injury due to the TFAM knockdown, culminating in apoptosis 19, and so to identify the cause of the apoptosis JC-1 staining was conducted. Culture cells in 6-well plates at a density of 5 × 10^5^ cells/ ml in a CO_2_ incubator overnight at 37 °C. 200μl of the JC-1 staining solution (JC-1 Mitochondrial membrane potential assay, Cayman, Ann Arbor, U.S.A) was added into each well. Samples were incubated in a CO_2_ incubator at 37°C for 30 minutes. After incubation, images were acquired using the BZ-X700 (KEYENCE, Tokyo, Japan). Viable cells were detected as red and apoptotic cells as green.

### Immunostaining for ATP synthase, 8-OHdG and HIF-1α

To confirm the loss of mitochondrial function, we stained for the presence/absence of adenosine triphosphate (ATP) production, which is a major component of mitochondrial function. Also, since oxidative injury has been implicated in the osteonecrosis animal model 2, we performed immunocytochemical studies of the oxidative stress marker 8-hydroxy-2'-deoxyguanosine (8-OHdG) as well as hypoxia inducible factor-1α (HIF-1α), an index of hypoxia. Cultured cells were fixed in 4 paraformaldehyde, washed in phosphate buffered saline (PBS), and permeabilized with 0.3% Triton X-100 in PBS. Nonspecific binding was blocked by incubating sections with 10% bovine serum albumin (Dako Cytomation, Santa Clara, CA) in PBS for 15 minutes. They were incubated with anti-ATP synthase (ATP5A) (Proteintech, IL), anti-8-OHdG (Abcam, Cambridge, U.K), and anti-HIF-1α (Abcam) antibody for 2 hours at a concentration of 10.0, 5.0, or 5.0 µg/ml, followed by a fluorescent-labeled secondary antibody (Alexa 488, Therno Fisher Scientific) and by DAPI for 30 minutes. After washing, a prolong diamond antifade mountant (Therno Fisher Scientific) was added, and cover slips were mounted. Images were acquired using Zeiss-LSM710. For ATP5A, cultured cells were labeled with the first antibody after labeling with the mitochondrial membrane potential-dependent probe.

### Western blotting

For quantification, immunoblotting for TFAM, 8-OHdG and HIF-1α was performed on MLO-Y4 cells. Proteins were extracted using protein extraction solution (PRO-PREPTM, iNtRON Biotechnology, Kyungki-Do, Korea). Extracted protein (20.0µg) was applied to and electrophoresed on a 10% polyacrylamide gel, and transferred to a nitrocellulose membrane (WAKO, Tokyo, Japan). The membranes were reacted overnight at 4 °C with anti-TFAM (RayBiotech, Norcross Georgia, U.S.A), anti-8-OHdG (ABBIOTEC, San Diego, CA), anti-HIF-1α (abcam) at a concentration of 1.0, 2.5, or 1.0 µg/ml. After incubation with peroxidase-labeled goat anti-rabbit or mouse IgG antibody (Dako Cytomation, Tokyo, Japan) for 1 hour at room temperature and vigorous washing, the nitrocellulose membrane was incubated with Chemiluminescence Luminol Reagent (Immuno Star LD, Wako, Tokyo, Japan) and photographed digitally using ImageQuant LAS 4000 mini (GE healthcare Japan Co, Tokyo, Japan). All samples were standardized by immunoblot using anti-actin mouse monoclonal antibody (Sigma Chemical Co., St. Louis, MO).

### Statistical analysis

All quantified results were expressed as the mean ± SD. Statistical significance in the comparison of apoptosis or necrosis between the control and each of the experimental groups was analyzed with Dunnett's multiple comparisons test. P values less than 0.05 were accepted as statistically significant. The statistical analysis was performed using StatView J-5.0 software (SAS Institute, Cary, USA).

## Results

### Expression of oxidative injury markers in glucocorticoid-induced osteocytic cells in hypoxia

**Figure 1:**
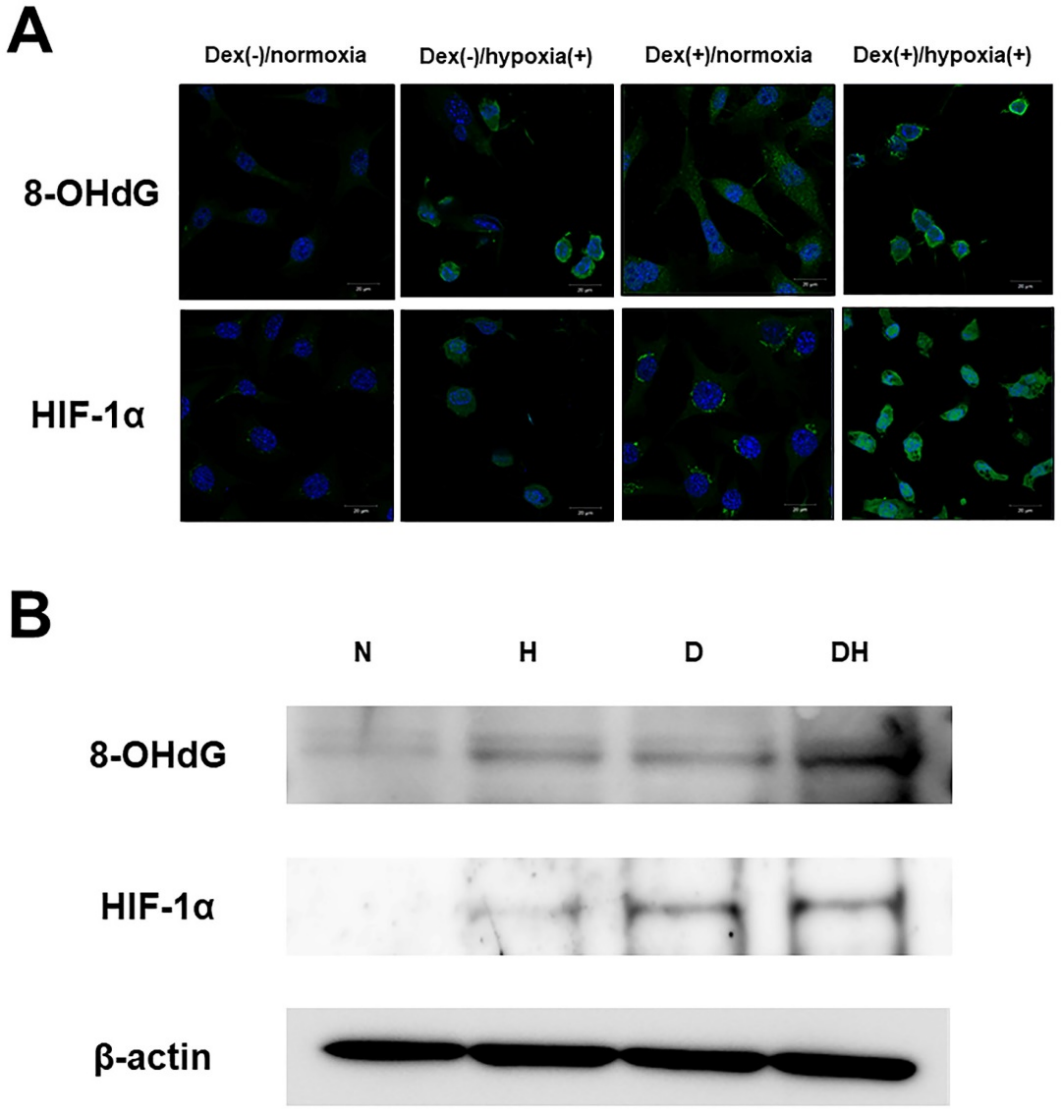
** Expression of oxidative stress and hypoxia markers. A.** Immunofluorescent staining of 8-OHdG and HIF-1α. In 20% O_2_, the group without addition of Dex was named Dex(-)/normoxia, that subjected to addition of Dex alone Dex(+)/normoxia, that with hypoxia alone Dex(-)/hypoxia(+), and that subjected to the combination of Dex and hypoxia Dex(+)/hypoxia(+). Regarding 8-OHdG, in Dex(-)/normoxia almost no expression was found, while in Dex(-)/hypoxia(+) and Dex(+)/normoxia expression was found in the cytoplasm. In Dex(+)/hypoxia(+) intense expression was found. Regarding HIF-1α, there was almost no expression in Dex(-)/normoxia, in Dex(+)/normoxia expression was limited to the cytoplasm, while in Dex(-)/hypoxia(+) expression was found in the nucleus, with transition within the nucleus seen. In Dex(+)/hypoxia(+) as compared with Dex(-)/hypoxia(+) expression was enhanced. Each five independent experiments were carried out. (Scale bar: 20 µm) **B.** Western blotting of 8-OHdG (36kDa) and HIF-1α (95-120 kDa). By Western blotting no obvious expression was found in either Dex(-)/normoxia (N). Both 8-OHdG and HIF-1α in Dex(-)/hypoxia(+) (H) and Dex(+)/normoxia (D) showed slight expression, and in Dex(+)/hypoxia(+) (DH) expression was enhanced. Each three independent experiments were carried out.

Immunofluorescent staining showed almost no 8-OHdG expression in Dex(-)/normoxia. In Dex(-)/hypoxia(+) and Dex(+)/normoxia expression was found in the cytoplasm, while in Dex(+)/hypoxia(+) intense expression was found. As for HIF-1α, there was almost no expression in Dex(-)/normoxia, in Dex(+)/normoxia expression was limited to the cytoplasm, while in Dex(-)/hypoxia(+) transition of HIF-1α into the nucleus was seen. In Dex(+)/hypoxia(+), as compared with Dex(-)/hypoxia(+) enhanced expression was found (**Figure 1A**). In WB too, both 8-OHdG and HIF-1α showed enhanced expression in Dex(+)/hypoxia(+) as compared to all of the other groups (**Figure 1B**).

### Mitochondrial functional injury by TFAM knockdown

**Figure 2:**
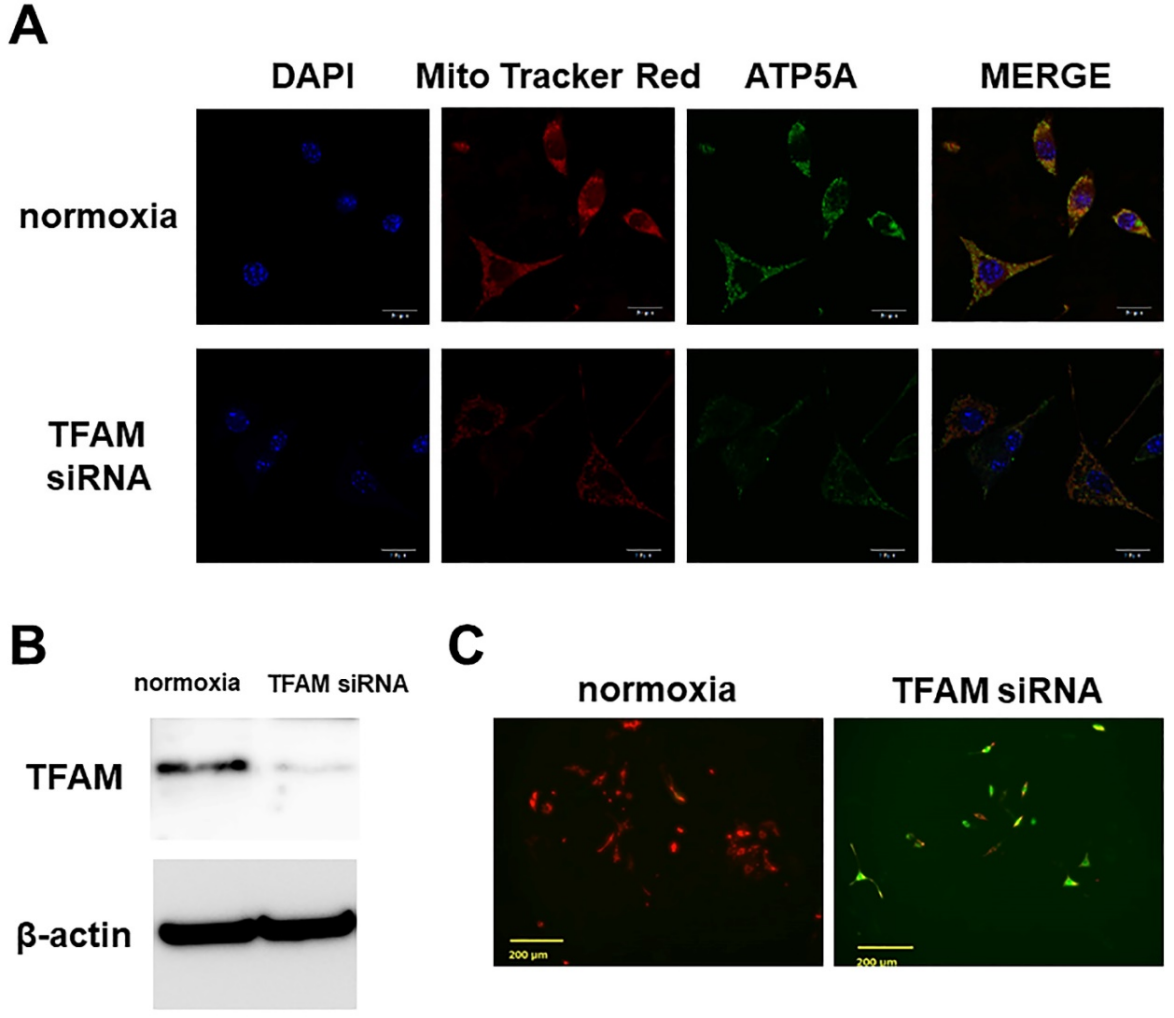
** Effects of TFAM knockdown using siRNA. A.** Mitochondrial membrane potential (Mito Tracker Red) and expression of ATP5A. ATP5A which documents the existence of ATP production, a major mitochondrial function, was detected. In Dex(-)/normoxia (normoxia) the mitochondrial membrane potential was preserved, and ATP5A expression was also found. By TFAM knockdown the mitochondrial membrane potential was no longer preserved, and ATP5A expression was attenuated. Each five independent experiments were carried out. (Scale bar: 20 µm) **B.** Western blotting of TFAM siRNA-transfected cells. In WB TFAM (29kDa) expression was significantly inhibited by TFAM siRNA. Each three independent experiments were carried out. **C.** JC-1 staining. On JC-1 staining, the mitochondrial membrane potential decreased by the osteocyte apoptosis induced by TFAM knockdown. Each three independent experiments were carried out. Viable cells: red, apoptotic cells: green. (Scale bar: 200 µm).

Immunostaining showed preservation in Dex(-)/normoxia of the mitochondrial membrane potential, and ATP5A expression. However, with TFAM knockdown, the membrane potential was no longer preserved, and ATP5A expression was attenuated (**Figure 2A**). In WB a significant inhibition of TFAM expression by siRNA was confirmed (**Figure 2B**). Also, with JC-1 staining we confirmed that a decrease in mitochondrial membrane potential was the factor underlying the osteocyte apoptosis induced by TFAM knockdown (**Figure 2C**).

### Inhibition of osteocytic cell necrosis by TFAM administration

**Figure 3:**
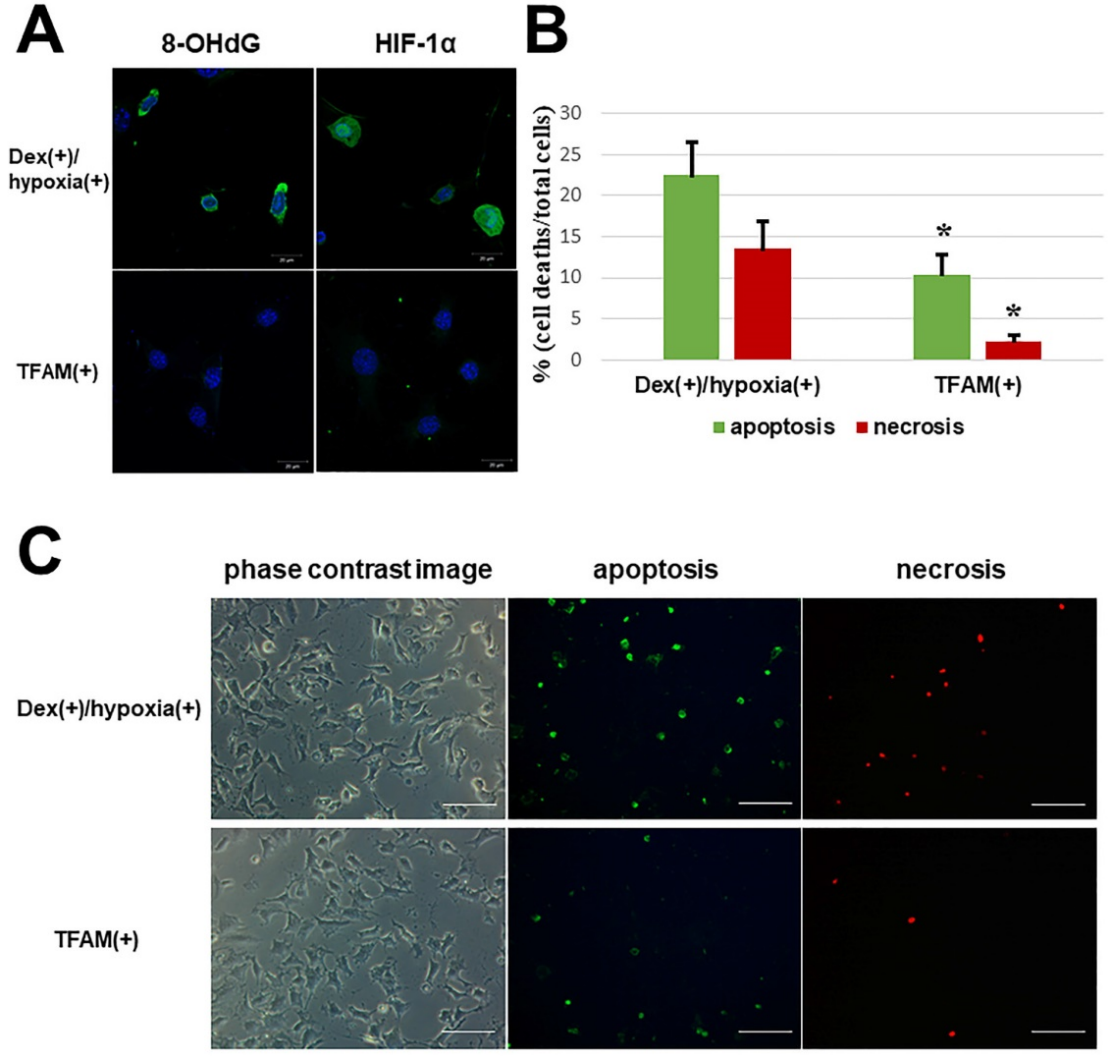
** Oxidative stress and hypoxia marker expression and number of osteocytic cell deaths after TFAM addition to osteocytes subjected to glucocorticoid administration in hypoxic environment.** TFAM was added to Dex(+)/hypoxia(+) and cultured for 24h (TFAM(+)). **A.** Immunofluorescent staining of 8-OHdG and HIF-1α with the addition of TFAM. With the addition of TFAM, the expression of both 8-OHdG and HIF-1α was inhibited. Each five independent experiments were carried out. (Scale bar: 20 µm) **B.** Graph indicates the percentages of apoptotic and necrotic cells in the indicated conditions. The numbers of apoptotic or necrotic cells were counted and related to the total number of cells. Columns and bars indicate means and S.D. respectively (n=5). **C.** Immunofluorescent staining of apoptotic and necrotic cells using an Apoptotic/Necrotic Cells Detection Kit as described in Materials and Methods. With addition of TFAM a significant decrease in the number of osteocytic cell deaths was noted as compared with Dex(+)/hypoxia(+) (*p<0.01). (Scale bar: 100 µm).

With the addition of TFAM, both 8-OHdG and HIF-1α in Dex(+)/hypoxia(+) showed attenuated expression (**Figure 3A**). Also, in contrast to the number of osteocytic cell deaths in Dex(+)/hypoxia(+) showing apoptosis (22.5 ± 4.0%) and necrosis (13.5 ± 3.4%), after the addition of TFAM both apoptotic cells (10.4 ± 2.4%), and necrotic cells (2.3 ± 0.7%) significantly decreased (*p<0.01) (**Figure 3B, C**).

## Discussion

Tissue hypoxia and tissue oxidative stress have been documented in earlier studies to play significant roles in the development of glucocorticoid-induced osteonecrosis 2. In hitherto conducted osteonecrosis-related research using osteocytes cultured in a hypoxic environment with addition of glucocorticoid, angiogenesis-osteogenesis coupling injury (A-O injury) *in vivo* has been reproduced in vitro, with a significant increase in osteocytic cell necrosis also documented 5. In the present investigation, the expression of 8-OHdG was enhanced in an environment the same as that of osteocytes. In this way, it was confirmed that severe oxidative injury could be induced just like in an osteonecrosis animal model subjected to hypoxia and glucocorticoid administration. Furthermore, it was confirmed from the state of HIF-1α expression that osteocytes are exposed to even greater hypoxic stress with the combination of hypoxia and glucocorticoids than with exposure to either stressor alone. Because HIF-1α suppresses the oxidative phosphorylation reaction by mitochondria, it is known to inhibit mitochondrial function and induce cell death 20-22. Namely, A-O injury itself is thought to cause cell death in osteocytes thereby creating a worst case scenario.

In this experiment, our attention was drawn to mitochondria that are very adversely impacted by diverse stresses including oxidative injury, and TFAM which plays roles in the preservation and repair of mitochondrial DNA. Under *in vivo* conditions, intraosseous TFAM has been shown to decrease following glucocorticoid administration 11. Since TFAM is originally present intracellularly, by knocking down TFAM with siRNA, the performance of TFAM in the osteocytic cells themselves could be confirmed. It has been suggested that exhaustion of the supply of TFAM in osteocytic cells threatens the preservation and survival of their mitochondria, with ATP production also ceasing. Namely, the TFAM present in osteocytic cells must be regarded as an essential factor for the preservation of mitochondria and cells.

In the heart failure model, inducing overexpression of cardiomyocyte TFAM has been reported to inhibit mitochondrial oxidative stress, and to improve cardiac function by suppressing pathologic cardiac hypertrophy 13,14. In the present experiment as well, by inducing overexpression of TFAM in osteocytic cells cultured in a hypoxic environment to which glucocorticoid was added, inhibition of oxidative injury and mitochondrial dysfunction were confirmed. In the osteonecrosis animal model, both oxidative injury and mitochondrial injury have been implicated 2,11. The results of the present study implicated oxidative injury in the creation of a state that culminated in the development of osteocytic cell necrosis. Moreover, since the addition of TFAM suppressed the development of osteocytic cell necrosis, we concluded that mitochondrial dysfunction is deeply implicated in this process. This means that to prevent osteocytic cell necrosis, inhibition of oxidative and hypoxic injuries due to stress, as well as minimizing the mitochondrial functional injury associated with these injuries are of great importance. At present, various experiments focusing on prevention are underway. From now on studies focusing on preservation of mitochondrial function too will be needed, while clinically strategies to help preserve mitochondrial function in osteocytes should also be actively pursued.

## Figures and Tables

**Table 1:** siRNA sequences

Gene accession	number siRNA sequence (5'-3')
TFAM, MSS278271	Sense, CACAGAACAGCUACCCAAAUUUAAA
	Anti-sense, UUUAAAUUUGGGUAGCUGUUCUGUG
TFAM, MSS278272	Sense, UACAAAGAAGCUGUGAGCAAGUAUA
	Anti-sense, UAUACUUGCUCACAGCUUCUUUGUA
TFAM, MSS278273	Sense, GGCAUAUAUUCAGCUUGCUAAAGAU
	Anti-sense, AUCUUUAGCAAGCUGAAUAUAUGCC

## Abbreviations

TFAM: mitochondrial transcription factor A
8-OHdG: 8-hydroxy-2'-deoxyguanosine
HIF-1α: hypoxia inducible factor-1α
ATP: adenosine triphosphate
α-MEM: α-minimal essential medium
PBS: phosphate buffered saline
A-O injury: angiogenesis-osteogenesis coupling injury
